# STK26 Promotes the Stabilization of ATF6 to Facilitate the Progression of Colorectal Cancer

**DOI:** 10.3390/ijms26168052

**Published:** 2025-08-20

**Authors:** Yuetian Ding, Jianwei Ren, Changwei Hu, Jiayue Han, Jin Zhang, Zhengsha Huang, Youfan Zhang, Weizhou Wang, Weizhe Yu, Qipeng Shu, Shangze Li

**Affiliations:** 1 School of Medicine, Chongqing University, Chongqing 400014, China; hcw_4125128@stu.xjtu.edu.cn (C.H.); 202337131095@stu.cqu.edu.cn (J.H.); 202237021058t@stu.cqu.edu.cn (J.Z.); 202337131080@stu.cqu.edu.cn (Y.Z.); 20243701006g@stu.cqu.edu.cn (W.W.); 2 College of Medicine, Tibet University, Lhasa 850000, China; ytding@stu.utibet.edu.cn (Y.D.); rjw@utibet.edu.cn (J.R.); 18886207417@163.com (Z.H.); 18037828477@163.com (W.Y.)

**Keywords:** CRC, UPR, tumorigenesis, STK26, ATF6

## Abstract

STK26 is highly expressed in colorectal cancer (CRC) and linked to tumorigenesis. Although implicated in unfolded protein response (UPR)-related oxidative stress, whether STK26 regulates CRC occurrence via the ATF6 pathway—a classic UPR branch governing proteostasis and cell survival—remains unestablished. In our research, we found that STK26 expression aberrantly upregulated in CRC is closely associated with poor prognosis. In vitro, tumor phenotype assays showed that STK26 drives CRC cell growth, proliferation, and migration. These effects were reversed by the ATF6 inhibitor Ceapin-A7, demonstrating that STK26’s oncogenic function depends on ATF6. Moreover, transcriptome sequencing revealed that STK26 is associated with the protein folding, sorting, and degradation pathway, and a luciferase reporter assay showed that STK26 activated the ATF6 signal pathway. Furthermore STK26 interacted with p50ATF6 and enhanced its protein stabilization. In vivo studies demonstrated that the administration of the STK26 inhibitor Hesperadin significantly suppressed CRC growth, suggesting a tumor-promoting role for STK26 in CRC pathogenesis. In summary, our research reveals that STK26 is a novel regulator that promotes the growth, proliferation, and migration of CRC cells by activating the ATF6 signaling pathway and stabilizing p50ATF6. Hence, the STK26-ATF6 axis has the potential to become a new target for treating colorectal cancer.

## 1. Introduction

Colorectal cancer (CRC) is a type of malignant tumor with high incidence and mortality rates among cancers [1]. Due to its high recurrence and metastasis characteristics, the prognosis of CRC patients remains poor, especially for those in the late stage [2]. Recently, research has found that oxidative stress plays an essential role in the occurrence and development of colorectal cancer [3]. Reports have suggested that biomarkers of oxidative stress could serve as valuable indicators of the early diagnosis of CRC patients [4,5]. When cells are exposed to various stress factors, such as nutrient deprivation and oxidative stress, the endoplasmic reticulum (ER) accumulates a large number of unfolded or misfolded proteins. This triggers the activation of a series of cellular signal pathways, a process known as the unfolded protein response (UPR) [6,7]. The UPR comprises three major signal pathways [8]: inositol-required enzyme 1 (IRE1), protein kinase-like ER kinase (PERK), and activating transcription factor 6α (ATF6α). Dysregulation of theses pathways, particularly ATF6α signaling, is closely associated with the occurrence of colorectal cancer [9].

Under ER stress, the 90 kDa ATF6 (p90) in the ER dissociates from GRP78/BiP and translocates to the Golgi apparatus, where it is cleaved by site-1 protease (S1P) within its luminal domain. Subsequently, the N-terminal portion of ATF6 is further cleaved by site-2 protease (S2P), releasing the 50 kDa cytosolic domain (p50). This p50ATF6 then translocates to the cell nucleus and activates the transcription of downstream genes [10].

Previous research has demonstrated that post translational modifications (PTMs) are essential regulators of ATF6 activity. PERK-mediated phosphorylation of eIF2α activates the PERK/eIF2α~P/ATF4 signaling pathway, thereby promoting the synthesis and nuclear translocation of 90 kDa ATF6 (p90) [11]. In research on bladder cancer, it was found that OTUB1 stabilizes 90 kDa ATF6 (p90) by inhibiting its ubiquitination, consequently activating the ATF6 signal pathway [12]. However, research on the stability of 50 kDa ATF6 (p50) is extremely limited. Only Hou et al. have found that SUMO1 and PIAS1 mediate SUMOylation of the 50 kDa ATF6 (p50), inhibiting its transcriptional activity [13]. In short, research on the other mechanisms mediating the stability of the 50 kDa ATF6 (p50) is limited. The potential roles of other PTMs, such as phosphorylation or ubiquitination, in regulating the 50 kDa ATF6 (p50) stability and activity still require further elucidation.

STK26 (MST4), a member of the mammalian Ste20-like (MST) kinases family, contains a conserved N-terminal kinase domain and a C-terminal regulatory domain [14]. Previous research suggests that STK26 is involved in regulating cell proliferation, apoptosis, development, cell polarity [15], and cell migration in cancers, such as prostate cancer [16], pancreatic cancer [17], liver cancer [18], glioblastoma [19], etc. These studies clearly reveal that STK26 is closely associated with tumorigenesis. Meanwhile, research also demonstrates that STK26 is involved in oxidative stress [20]; for example, under oxidative stress conditions, STK26 can regulate the immune inflammatory response by directly phosphorylating TRAF6 [21]. Interestingly, UPR in cancer cells requires maintenance of various stresses (including oxidative stress) [22,23], and the UPR signaling pathway is closely linked to oxidative stress in tumor cells [24]. However, whether SKT26 regulates tumorigenesis via UPR remains unknown.

Here, transcriptome sequencing revealed that STK26 is associated with UPR, furthermore the expression of STK26 was upregulated and positively associated with ATF6 and its downstream effectors. We then verified that STK26 is a novel regulator of the 50 kDa ATF6 (p50) and prompts its stabilization by interacting with the 50 kDa ATF6 (p50), posing an important impact on the occurrence of colorectal cancer. In summary, we found a new mechanism of the 50 kDa ATF6 (p50), regulated by STK26, in the development and progression of colorectal cancer.

## 2. Results

### 2.1. STK26 Is Aberrantly Overexpressed in Colorectal Cancer

**Figure 1:**
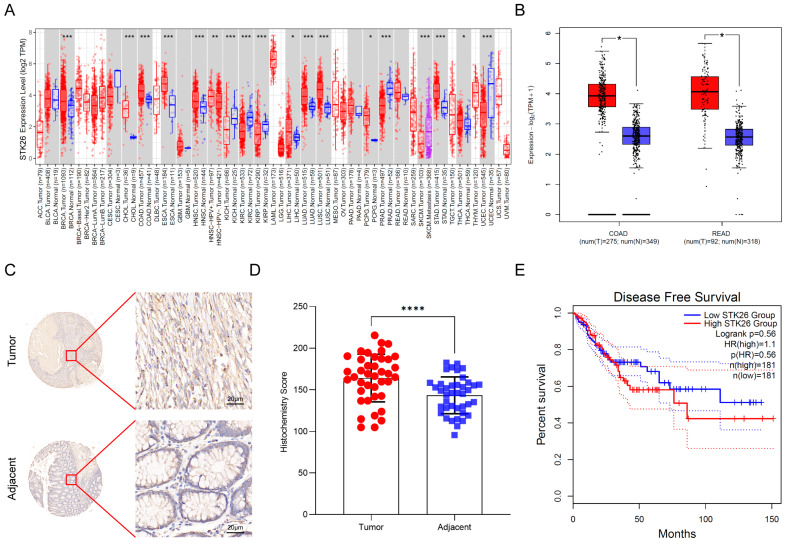
STK26 is aberrantly overexpressed in colorectal cancer: (**A**) The STK26 expression level in pan-cancers on TIMER2.0 (http://timer.cistrome.org/ (accessed on 13 August 2025)). (**B**) STK26 expression in colon and rectal cancer tissues (red) (*n* = 275 and *n* = 92, respectively) compared with those non-cancerous tissues (blue) (*n* = 349 and *n* = 318, respectively) obtained from the GEPIA 2 online database (http://gepia2.cancer-pku.cn/#index (accessed on 13 August 2025)). TIMER2.0 compares TCGA tumors to adjacent normals, while GEPIA 2 uses TCGA tumors and non-cancerous tissues from both GTEx- and TCGA-adjacent normals. (**C**) IHC staining for STK26 expression in colorectal cancer tissue microarrays containing tumor and adjacent tissues. (**D**) Analysis of STK26 expression in colorectal tissue microarrays from tumor and adjacent tissues. (**E**) Disease-free survival of patients with low and high STK26 expression. * *p* < 0.05, ** *p* < 0.01, *** *p* < 0.001, **** *p* < 0.0001.

To investigate STK26 expression patterns across cancers, we first analyzed the expression of STK26 in pan cancer using the TIMER2.0 online website and found that STK26 is upregulated in colon cancer (Figure 1A). Then, to assess whether STK26 is aberrantly overexpressed in colorectal cancer, the GEPIA 2 online tool was used. The results showed that compared to those non-cancerous samples, the mRNA expression level of STK26 was significantly upregulated in colorectal cancer patients (Figure 1B). To further validate these findings, immunohistochemical analysis of 40 paired colorectal cancer and adjacent normal tissues confirmed significantly higher STK26 protein expression in tumor tissues compared to matched normal counterparts (Figure 1C,D). Critically, the higher mRNA expression of STK26 correlated with worse disease-free survival in colorectal cancer patients (Figure 1E). Therefore, these findings indicate that the expression of STK26 is upregulated in colorectal cancer, and higher STK26 expression is associated with poor clinical outcomes.

### 2.2. Overexpressing STK26 Promotes CRC Cells Growth, Proliferation, and Migration

**Figure 2:**
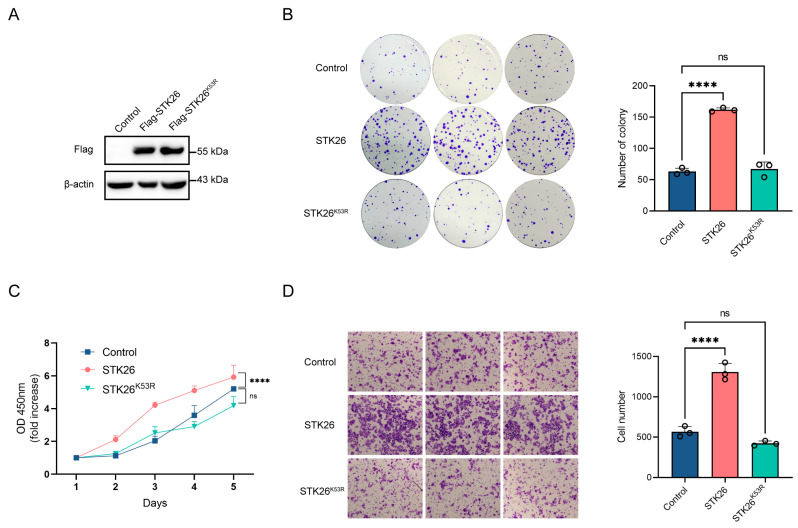
Overexpression of STK26 promotes CRC cells growth proliferation and migration: (**A**) STK26-OE was identified by immunoblotting with HCT116 cells. (**B**) The viability of STK26-OE HCT116 cells was determined by a colony formation experiment. The number of colonies was recorded (*n* = 3). (**C**) The cell proliferation of STK26-OE HCT116 cells was determined by a Cell Counting Kit-8 (CCK8) assay (*n* = 6). (**D**) The cell migration ability of STK26-OE HCT116 cells was determined by transwell experiments (*n* = 3). ns, no significant difference. **** *p* < 0.0001.

To investigate the biological role of STK26 in colorectal cancer development, the mRNA and protein expression of STK26 in CRC cells, including SW48, SW480, HCT15, HCT116, DLD1, and LoVo cells, were detected via qPCR and Western blot. These analyses indicated that STK26 is highly expressed in SW480 cell lines and relatively low in HCT116 cell lines (Supplementary Figure S1A,B). Subsequently, we constructed the stable STK26 and its kinase inactive K53R mutant (STK26^K53R^) [25] overexpression cell lines in HCT116 cells, and the expressions of STK26 and STK26^K53R^ were verified by Western blot (Figure 2A). Then, a series of in vitro tumor biology assays were performed. The colony formation and CCK-8 assays showed that ectopically expressed STK26—not the STK26^K53R^ mutant—significantly increased cell viability and colony number compared to the control group (Figure 2B,C). Additionally, the transwell migration assay demonstrated a similar result in that the overexpression of STK26 markedly enhanced the migration ability of colorectal cancer cells (Figure 2D). Therefore, these results indicate that STK26 significantly promotes the growth, proliferation, and migration of colorectal cancer cells, and these effects are dependent on its kinase activity.

### 2.3. STK26 Deficiency Represses CRC Cells Growth, Proliferation and Migration

**Figure 3:**
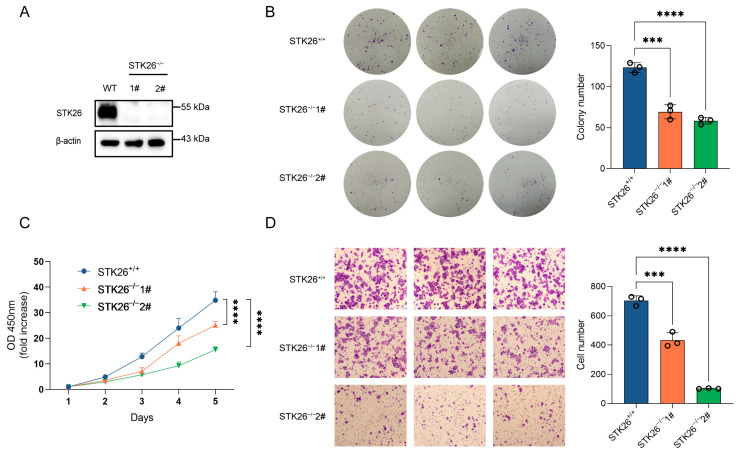
STK26 deficiency represses CRC cells growth, proliferation, and migration: (**A**) STK26 knockout was identified by immunoblotting with SW480 cells. (**B**) Colony formation experiment was employed to measure the viability of WT and STK26-deficient SW480 cells, and the number of clone was counted (*n* = 3). (**C**) Cell proliferation ability of WT and STK26-deficient SW480 cells was detected with a Cell Counting Kit-8 (CCK8) assay (*n* = 6). (**D**) Cell migration ability of WT and STK26-deficient SW480 cells was measured by transwell experiments (*n* = 3). *** *p* < 0.001, **** *p* < 0.0001.

To further elucidate the role of STK26 in colorectal cancer, STK26-deficient cell lines were constructed in SW480 cells by CRISPR/Cas9 gene editing technology, which was confirmed by Western blotting (Figure 3A). The colony formation assay demonstrated that the number of colonies formed in STK26-deficient SW480 cells was markedly reduced, and the size of colonies was smaller (Figure 3B); moreover, the CCK-8 assay showed that the proliferative capacity of STK26-deficient SW480 cells was significantly decreased compared to the wild-type (Figure 3C). Furthermore, the transwell migration assay revealed that the migratory ability of STK26-deficient SW480 cells was diminished compared to the wild-type cells (Figure 3D). While minor quantitative differences existed between clones “#1” and “#2”, potentially reflecting CRISPR editing heterogeneity, compensatory adaptation, or stochastic single-cell effects, both exhibited unequivocal suppression of oncogenic phenotypes, establishing STK26 as a key regulator of colorectal cancer progression. In brief, these data indicate that the depletion of STK26 suppresses the growth, proliferation, and migration of colorectal cancer cells.

### 2.4. STK26 Deficiency Leads to Downregulation of the ATF6 Pathway

**Figure 4:**
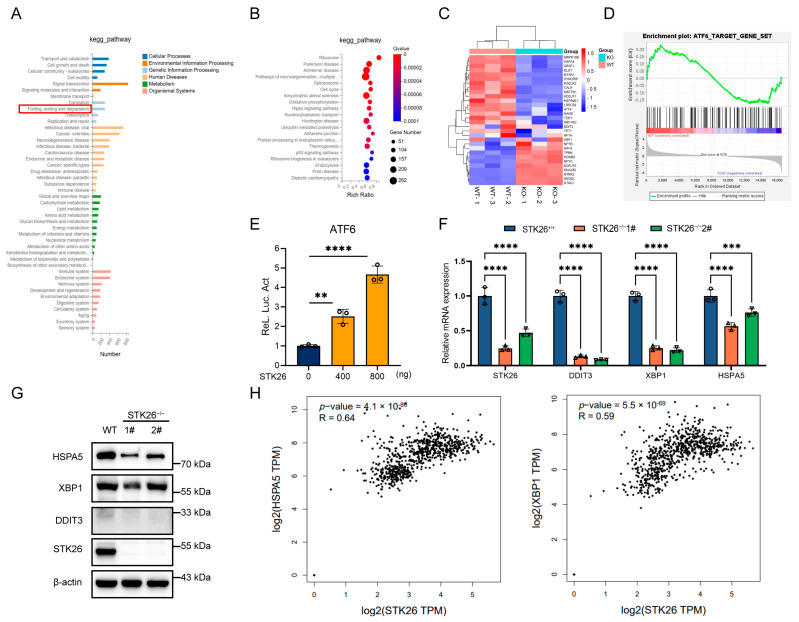
STK26 deficiency leads to downregulation of the ATF6 pathway: (**A**) KEGG pathway enrichment analysis of the different genes expressed between WT and STK26-deficient SW480 cells. (**B**) Bubble diagram showing the enrichment of differentially expressed genes in the biological process category. (**C**) Heatmap analysis of ATF6 downstream genes in WT and STK26-deficient SW480 cells. (**D**) GSEA of ATF6 signal pathway target genes in WT and STK26-depleted SW480 cells. (**E**) STK26-enhanced ATF6 activation, as shown by a luciferase reporter assay in HEK293T cells (*n* = 3). (**F**) The mRNA level of genes with ATF6-trigered transcription in STK26-deficient SW480 cells were determined by RT-qPCR analysis (*n* = 3). (**G**) The protein level of genes with ATF6-trigered transcription in STK26-deficient SW480 cells were determined by immunoblot analysis (*n* = 3). (**H**) The positive correlation between STK26 and ATF6 target genes from the GEPIA 2 online tool. ** *p* < 0.01, *** *p* < 0.001, **** *p* < 0.0001.

To elucidate the specific mechanisms by which STK26 regulates the development of colorectal cancer, we performed eukaryotic transcriptome sequencing in wild-type and STK26-deficient SW480 cells. The KEGG pathway analysis showed that folding, sorting and degradation of protein was obviously enriched in STK26-deficient SW480 cells (Figure 4A), and the bubble diagram also indicates that Parkinson’s disease and Alzheimer’s disease, which are closely correlative with UPR [26,27], are clearly intensified (Figure 4B). Accordingly, we hypothesized that STK26 highly expressed in the Golgi apparatus may be linked to the folding of protein or UPR. To verify this, the heatmap and GSEA analysis indicated that STK26 is bound up with the ATF6 signal pathway (Figure 4C,D). Moreover, the luciferase reporter assay also showed that STK26 could activate the ATF6 signal pathway (Figure 4E). Then, to further verify our hypothesis, we measured the mRNA expression of downstream genes in the ATF6 signal pathway, which revealed that downstream genes, including DDIT3, HSPA5, and XBP1 exhibited significantly reduced mRNA levels (Figure 4F). Meanwhile, the protein levels of ATF6 downstream showed equivalent results, which indicated that STK26 regulates the activity of the ATF6 signal pathway (Figure 4G). Moreover, analysis of TCGA colorectal cancer data using the GEPIA2 online tool revealed that STK26 exhibits significant positive correlations with mRNA expression levels of ATF6 pathway downstream effectors (HSPA5, XBP1), clinically corroborating our experimental findings (Figure 4H). To sum up, these data demonstrate a functional link between STK26 and the ATF6 signal pathway in colorectal cancer.

### 2.5. STK26 Interacts with and Stabilizes ATF6

**Figure 5:**
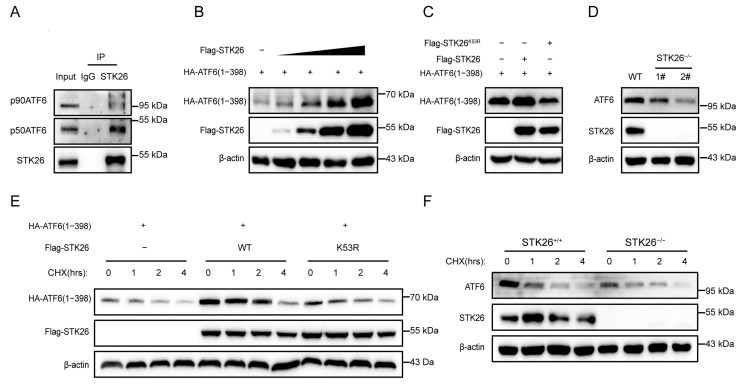
STK26 interacts with and stabilizes ATF6: (**A**) The interaction of endogenous STK26 and ATF6 was examined by co-immunoprecipitation in SW480 cells. (**B**) The protein level is found to be positively correlated with HA-ATF6 (1−398aa) and increasing Flag-STK26 protein (0, 200 ng, 400 ng, 800 ng and 1 μg) in HEK293T cells by immunoblot assay. (**C**) The effect of STK26 or the STK26^K53R^ mutant on ATF6 protein levels in HEK293T cells. (**D**) The protein level of ATF6 in STK26-deficient SW480 cells. (**E**) The stability of ATF6 in the presence of STK26 or the STK26^K53R^ mutant after treatment with CHX (50 μg/mL) at the indicated time points. (**F**) The stability of ATF6 in the absence of STK26 after treatment with CHX (50 μg/mL) at the indicated time points.

Since ATF6 is a crucial member in the ATF6 signal pathway, we investigated their interactive relationship. The endogenous co-immunoprecipitation in SW480 cells showed that STK26 interacted with both the 90 kDa ATF6 (p90) and the 50 kDa ATF6 (p50), and the interaction of the latter was stronger (Figure 5A). Given that during ER stress, the 50 kDa ATF6 (p50) is the main form that translocated into the nucleus to function as a transcription factor [9], we constructed an HA-tagged 50 kDa ATF6 (p50) plasmid. Then, we co-transfected Flag-STK26 (0, 200 ng, 400 ng, 600 ng, and 800 ng) and HA-ATF6 (1−398) into HEK293T cells to evaluate the effect of STK26 on the stability of ATF6. The results showed that Flag-STK26 stabilized ATF6 in a dose-dependent manner (Figure 5B). To determine whether the stabilization of ATF6 by STK26 is related to its enzymatic activity, we co-transfected Flag-STK26 or Flag-STK26^K53R^ and HA-ATF6 (1−398) into HEK293T cells, and the results showed that the stabilization of ATF6 requires the enzymatic activity of STK26 (Figure 5C). Next, we evaluated the effect of STK26-depletion on the protein level of ATF6, which indicated that the knockout of STK26 in the cell line reduced the expression level of ATF6 (Figure 5D). Moreover, a cycloheximide chase (CHX) assay was performed to verify that ATF6 protein levels were regulated by STK26; the exogenous CHX experiment showed that STK26, instead of STK26^K53R^, significantly prolonged the half-life of ATF6 (Figure 5E), and the endogenous CHX assay also showed similar results (Figure 5F). Consequently, these data indicate that STK26 stabilizes and interacts with ATF6.

### 2.6. STK26 Promotes CRC Cells Growth, Proliferation, and Migration in an ATF6-Dependent Manner

**Figure 6:**
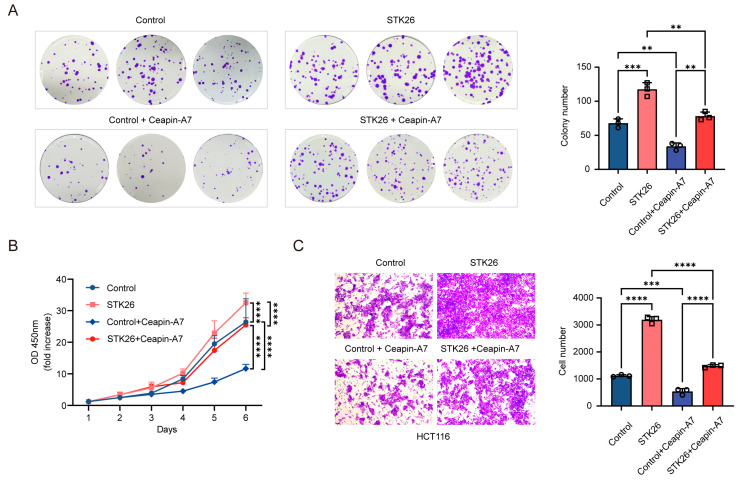
STK26 promotes CRC cells growth, proliferation, and migration in an ATF6-dependent manner: (**A**) The viability of the control group, control + Ceapin-A7 group, STK26 overexpression group, and STK26 overexpression + Ceapin-A7 group HCT116 cells were determined by a colony formation experiment. The number of colonies was recorded (*n* = 3). (**B**) The cell proliferation of the control group, control + Ceapin-A7 group, STK26 overexpression group, and STK26 overexpression + Ceapin-A7 group HCT116 cells were determined by a Cell Counting Kit-8 (CCK-8) assay (*n* = 6). (**C**) The cell migration ability of the control group, control + Ceapin-A7 group, STK26 overexpression group, and STK26 overexpression + Ceapin-A7 group HCT116 cells were determined by transwell experiments (*n* = 3). The data are presented as mean ± SEM from three independent experiments. ** *p* < 0.01, *** *p* < 0.001, **** *p* < 0.0001.

As we know, STK26 promotes the tumorigenesis of CRC and the stabilization of the ATF6 protein. To determine whether STK26’s effects on CRC cells growth, proliferation, and migration are dependent on ATF6, we utilized the ATF6 inhibitor Ceapin-A7 to experimentally verify this hypothesis. Ceapin-A7 is a specific inhibitor of ATF6 that exerts inhibitory effects by competitively binding to ATF6 and preventing its translocation to the Golgi apparatus [28]. The colony formation assay (Figure 6A) indicated that Ceapin-A7 significantly impaired the proliferative capacity and survival of HCT116 cells, reducing both colony number and size. This suggests that ATF6 inhibition disrupts cell-autonomous growth under low-density conditions. Notably, STK26 overexpression restored colony formation, rescuing both proliferative potential and clonogenic survival, thereby highlighting its role in compensating for ATF6-dependent growth regulation. Consistent with the colony formation results, the growth curve of CCK-8 (Figure 6B) showed that while STK26 overexpression enhanced HCT116 cell growth, this effect was significantly diminished by ATF6 inhibitor treatment. Furthermore, Ceapin-A7 could inhibit the migration of HCT116 cells, while overexpressing STK26 would offset the effect of the ATF6 inhibitor (Figure 6C). In summary, these results indicate that the promotion of CRC cells’ growth, proliferation, and migration by STK26 depends on the ATF6 signal pathway.

### 2.7. Hesperadin Effectively Inhibits the Growth of CRC In Vivo

**Figure 7:**
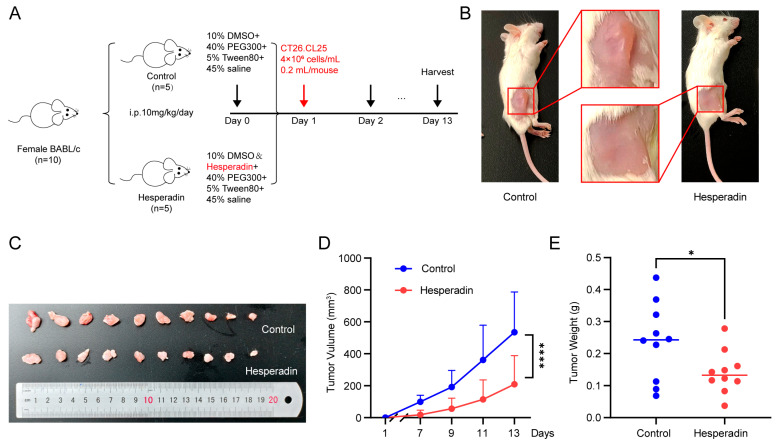
Hesperadin inhibits syngeneic tumor growth in vivo: (**A**) A total of 10 BALB/c mice were randomly divided into two groups and treated with Hesperadin (10 mg/kg/day, i.p.) for 13 days. (**B**,**C**) Image depicting subcutaneous tumor formation in mice after injection of 4 × 10^6^ cells/mL CT26.CL25 cells, with or without Hesperadin treatment. (**D**) Changes in tumor volume during Hesperadin treatment. Tumor volume was measured every 2 days. (**E**) The weights of resected tumors from each mouse. The data are presented as mean ± SEM from three independent experiments. * *p* < 0.05, **** *p* < 0.0001.

Research has shown that Hesperadin, originally described as an Aurora kinase inhibitor, reduces effectively the activity of STK26 at nanomolar concentrations [29]. To evaluate the impact of STK26 on tumorigenesis in vivo, this study established a homologous tumor transplantation model with CT26.CL25 cells based on this process (Figure 7A). As demonstrated in Figure 7B,C, Hesperadin treatment resulted in significantly smaller tumors compared to the control group. Longitudinal monitoring of tumor volume revealed that Hesperadin effectively attenuated tumor growth throughout the experimental period (Figure 7D). Consistent with these observations, the final tumor weights in the Hesperadin-treated group were also markedly reduced (Figure 7E). Therefore, these results show that compared with the control group, the Hesperadin treatment group significantly inhibited tumor growth, indicating that STK26 promoted the tumorigenesis of CRC in vivo.

## 3. Discussion

ATF6, a type-II transmembrane protein that contains an alkaline leucine zipper transcription factor in its cytoplasmic domain, is essential to tumorigenesis [30]. When misfolded proteins are detected in ER, ATF6 shuttles to the Golgi apparatus, where it is cleaved by site-1 and site-2 proteases to release the p50ATF6 (N) as a transcription factor. Then, the p50ATF6 is translocated to the nucleus to regulate related gene expression [31]. For this reason, the regulation of p50ATF6 is essential to control its downstream genes’ expression and tumorigenesis. Previous research has found the SUMOylation of p50ATF6 will repress the transcriptional activity of ATF6 [13]; here, we demonstrate that STK26, highly expressed in CRC, is a new regulator of p50ATF6.

STK26 is highly expressed in colorectal cancer and correlates with poor prognosis and survival rate in colorectal cancer patients. A tumor phonotype experiment in WT and STK26-deficient SW480 cells has enriched the function of STK26 in facilitating the growth, proliferation, and migration of tumor cells [32]. Further, eukaryotic transcriptome sequencing in wild-type and STK26-deficient SW480 cells reveals the significance of the folding, sorting, and degradation of protein pathway. In addition, previous research has found that STK26 can promote pituitary tumor development by regulating cell proliferation and survival in a low-oxygen microenvironment, and the activation of HIF-1 and its downstream nuclear targets is also key to the role of STK26 [33]. Currently, an increasing number of studies indicate that UPR and CRC are closely related [34]. So, we dedicated our research to the relationship between STK26 and UPR. In the process, we found that the ATF6 signal pathway is more closely related to the occurrence of CRC. Heatmap and GSEA analysis also confirmed that ATF6-related genes were downregulated in STK26-deficient SW480 cells. Then, qPCR and Western blotting demonstrated that the downstream effectors of ATF6 were also downregulated in STK26-deficient SW480 cells. What is more, we also found that STK26 could interact with p50ATF6 and regulate its stability dependent on its kinase activity. In vivo studies demonstrated that the administration of the STK26 inhibitor Hesperadin significantly suppressed CRC growth, suggesting a tumor-promoting role of STK26 in CRC pathogenesis.

**Figure 8:**
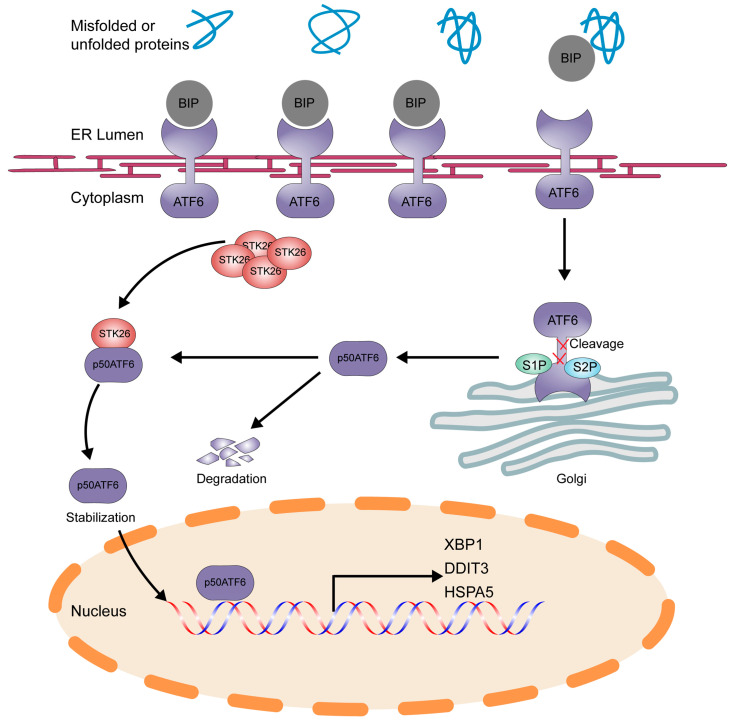
Schematic model of STK26-mediated stabilization of ATF6 in colorectal cancer. p50ATF6 was formed after being cleaved by the S1P and S2P enzymes in Golgi, and p50ATF6, in the process, will be degraded in the nucleus, while p50ATF6 will be stabilized by STK26, depending on its kinase activity.

Overall, our research identifies STK26 as a new regulator of p50ATF6 (Figure 8). Moreover, we also elucidate how STK26, highly expressed in CRC, facilitates tumorigenesis via the ATF6 signal pathway, which provides a new perspective on the role of STK26 in colorectal cancer. Targeting the STK26-p50ATF6 axis may be a new therapy for treating colorectal cancer. However, further research is still to be completed. Although we have found that the regulation of ATF6 by STK26 depends on its kinase activity, it remains unclear whether STK26-mediated phosphorylation occurs during this regulatory process. If so, the specific phosphorylation site of STK26 targeting ATF6 still needs to be explored. Since the p50ATF6 will not be activated persistently, even though STK26 prompts the stabilization of p50ATF6, how p50ATF6 is degraded remains to be elucidated. All these factors are still undergoing research and will be exhibited in our future work. Moreover, in our study, we found that STK26 may be related to oxidative phosphorylation. Previous studies have shown that STK26 plays a vital role in oxidative stress, so the role of STK26 in this field will be the focus of our future research.

## 4. Materials and Methods

### 4.1. Antibodies and Plasmids

The antibodies used in this research are as follows: anti-ATF6 antibody (#65880S, CST), anti-STK26 antibody (A20964, ABclonal, Woburn, MA, USA), anti-DDIT3 antibody (A20987, ABclonal), anti-HSPA5 antibody (A23453, ABclonal), anti-XBP1 antibody (A1731, ABclonal), anti-HA antibody (AE105 and AE008, ABclonal), anti-Flag antibody (AE005, ABclonal), and anti-β-actin antibody (60008-1-Ig, Proteintech, Rosemont, IL, USA). Plasmids were constructed per the Molecular Cloning Technical Guide [35] with the following key specifications: pHAGE-3 × Flag-STK26—BamH I and Sal I-linearized vector and STK26 ORF (NM_001042452.1); pHAGE-3 × Flag-STK26K53R—constructed through site-directed mutagenesis; and pcDNA5-HA-ATF6(1−398)—BamH I and Hind III-digested vector and ATF6 ORF (NM_007348.4). All inserts were amplified from SW480 cDNA. PCR primers are listed in Supplementary Table S1.

### 4.2. Cell Culture and Cell Lines

SW480, HCT116, SW48, HCT15, DLD1, and LoVo human colorectal cancer cell lines, as well as HEK293T and CT26.CL25 cells, were cultured in McCoy’s 5A or DMEM or RPMI 1640 supplemented with 10% FBS and 1% penicillin–streptomycin. Cells were incubated at 37 °C in a 5% CO_2_ incubator. All cells were obtained from ATCC. No mycoplasma contamination was detected.

### 4.3. Animal Experiment

Six-week-old female BALB/c mice, weighing approximately 15 g, were purchased from Jiangsu GemPharmatech Co., Ltd (Nanjing, China). All mice were housed in the laboratory of the Neuro-intelligence Center at Chongqing University under pathogen-free conditions. The animal experimental procedures strictly follow the guidelines for the care and use of experimental animals issued by the National Institutes of Health (NIH) in the United States and have been approved by the Chongqing University Experimental Animal Welfare Ethics Committee (IACUC number: CQU-IACUC-RE-202501-002). The specific steps are as follows: mice were randomly divided into a control group and a Hesperadin (HY-12054, MCE, Shanghai, China) group (*n* = 5), and CT26.CL25 cell suspension (4 × 10^6^ cells/mL, 0.2 mL/mouse) was subcutaneously implanted bilaterally into the dorsum. Starting one day before tumor cell implantation, intraperitoneal injection was administered. The control group was given a solvent (10% DMSO + 40% PEG300 + 5% Tween80 + 45% physiological saline), while the Hesperadin group was given a drug containing a solvent (10% DMSO dissolved Hesperadin + 40% PEG300 + 5% Tween80 + 45% physiological saline), with a dosage of 10 mg/kg/day until the end of the experiment. Tumor monitoring shows that local invasive lesions can form 7 days after tumor cell implantation. From the formation of the tumor, every 2 days, we used a vernier caliper to measure the tumor’s long diameter (a) and short diameter (b) and calculated the volume according to the following formula: V = 0.5ab^2^. When the tumor grows to a volume of ≤1000 mm^3^, the mice are euthanized via the cervical dislocation method. At the end of the experiment, we euthanized the mice, completely removed the tumor tissue, and measured the tumor weight.

### 4.4. Tumor Phenotype Analysis

Tumor phenotype analysis was performed as previously described [36]. For proliferation assays, cells were seeded at a density of 1000 cells per well in a 96-well plate. To measure cell viability, 1/10 volume of the CCK-8 reagent was added to each well and incubated at 37 °C for 1 h. Then, the absorbance at 450 nm was measured using a microplate reader. For the colony formation assays, cells were seeded at a density of 400–600 cells per well in a 6-well plate. After 2 weeks, the cells were stained with crystal violet, and the number of colonies formed was counted. For the transwell invasion assays, cells were seeded at a density of 5 × 10^4^ cells per well in the upper chamber of a transwell insert in serum-free medium. The lower chamber was filled with culture medium containing 20% FBS. After 48 h of incubation, the cells that had migrated through the membrane were stained with crystal violet. The number of migrated cells was counted under an inverted microscope. For phenotype experiments with the addition of ATF6 inhibitors, cells were pretreated with 6 μM Ceapin-A7 (HY-138450, MCE) for 24 h prior to experimental assays. The Ceapin-A7 was prepared in DMSO (final concentration ≤ 0.1%) and diluted in complete culture medium. Control groups received vehicle (DMSO) alone at equivalent concentrations. All other experimental conditions remained unchanged.

### 4.5. RNA Isolation for Real-Time Quantitative PCR

The cells were harvested with RNAiso Plus (9108, Takara, Japan) reagent, and total RNA was extracted according to the manufacturer’s instructions. cDNA was obtained with a reverse transcription kit (PR092S, Takara, Japan). The sequences of primers used are presented in Supplementary Table S1. Real-time quantitative PCR (RT-qPCR) was performed using SYBR Green PCR Master Mix (PR820A, Takara, Japan) with BIO-RAD CFX Real Time system.

### 4.6. Western Blot Analysis

The transfected cells were lysed in SDS loading buffer containing 2.5 mM Tris-HCl, pH 6.8, 2% SDS, and 8–12% glycerol. The samples were heated at 95 °C for 15 min. Total protein (20–40 μg) per lane was separated on a 10% SDS-PAGE gel and then transferred to a PVDF membrane, followed by 5% non-fat milk at room temperature for 1 h. Then, the membrane was incubated with the primary antibody overnight at 4 °C. The next day, the membrane was incubated with the secondary antibody for 1 h at room temperature before visualization in a Tanon 5200 Image System (Shanghai, China).

### 4.7. Co-Immunoprecipitation

SW480 cells were lysed using lysis buffer (20 mM Tris-HCl, pH 7.4, 150 mM NaCl, and 0.5% Triton X-100) supplemented with a protease and phosphatase inhibitor cocktail. The cell lysates were incubated with an appropriate amount of protein A/G agarose beads and 1–3 μg of the targeting antibody at 4 °C overnight with gentle rotation. After incubation, the beads were washed three times, and the bound proteins were eluted and separated by SDS-PAGE.

### 4.8. Transcriptome Sequencing

The wild-type and STK26-deficient SW480 cells were subjected to eukaryotic transcriptome sequencing, which was performed by BGI (Shenzhen, China). The differentially expressed genes were analyzed with KEGG and GSEA databases to identify enriched signaling pathways and biological processes. Fisher’s exact test was used to select the most significant pathways.

### 4.9. Luciferase Reporter Assay

After the HEK293T cells were seeded into 24-well plates, the luciferase reporter plasmid ATF6, pPL-CMV, and an increasing amount of Flag-STK26 were transfected into the HEK293T cells in each well. After 48 h, the luciferase reporter experiment was carried out using a dual-specific luciferase assay kit (DL101, Vazyme, China) on Promega.

### 4.10. Statistical Analysis

The experimental data was analyzed using GraphPad Prism 9.0 software. The results are presented as the mean ± SEM. For comparisons between two groups, Student’s *t*-test and analysis of variation (ANOVA) were employed to determine the statistical significance. *p* < 0.05 was statistically significant.

## Data Availability

The original contributions presented in this study are included in the article/Supplementary Material. Further inquiries should be directed to the corresponding authors.

## Supplementary Materials

The following supporting information can be downloaded at https://www.mdpi.com/article/10.3390/ijms26168052/s1.
