# Supplementary figures and images for: STK26 Promotes the Stabilization of ATF6 to Facilitate the Progression of Colorectal Cancer
Source: Int J Mol Sci. 2025 Aug 20;26(16):8052. doi: 10.3390/ijms26168052 (PMC12386624; doi:10.3390/ijms26168052)

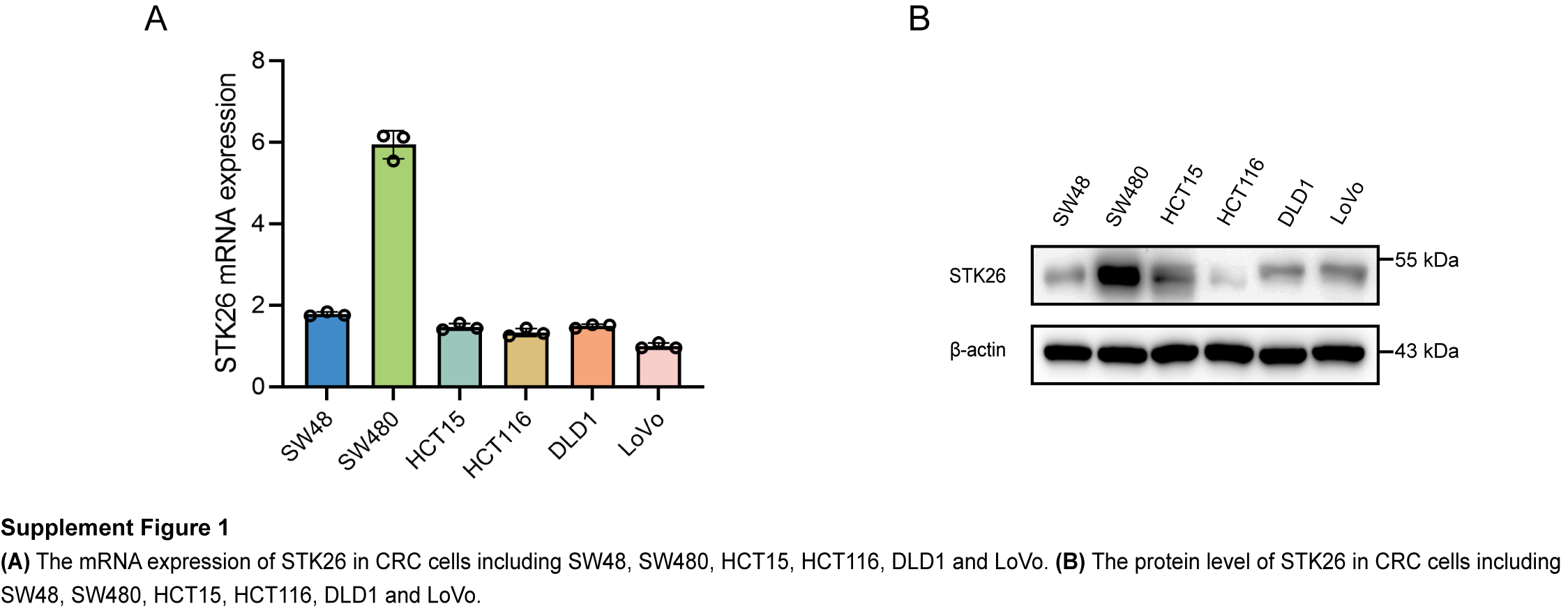

Supplement: Supplementary file 1 [file ijms-26-08052-s001.zip › Figure S1.tif]
